# Multicenter, randomised, open-label, non-comparative phase 2 trial on the efficacy and safety of the combination of bevacizumab and trabectedin with or without carboplatin in women with partially platinum-sensitive recurrent ovarian cancer

**DOI:** 10.1038/s41416-019-0584-5

**Published:** 2019-09-20

**Authors:** Nicoletta Colombo, Eleonora Zaccarelli, Alessandra Baldoni, Simona Frezzini, Giovanni Scambia, Eleonora Palluzzi, Germana Tognon, Andrea A. Lissoni, Daniela Rubino, Annamaria Ferrero, Gabriella Farina, Emanuele Negri, Angela Pesenti Gritti, Francesca Galli, Elena Biagioli, Eliana Rulli, Davide Poli, Chiara Gerardi, Valter Torri, Roldano Fossati, Maurizio D‘Incalci

**Affiliations:** 1 Istituto Europeo di Oncologia, Milano, Italy; 2 Università di Milano-Bicocca, Milano, Italy; 3 Istituto Oncologico Veneto, IOV-IRCCS, Padova, Italy; 4 Fondazione Policlinico Universitario A. Gemelli IRCCS Università Cattolica, Roma, Italy; 5 UO Ostetricia e Ginecologia - ASST degli Spedali Civili di Brescia, Università degli Studi, Brescia, Italy; 6 Università di Milano-Bicocca, Clinica Ostetrica e Ginecologica, Milano, Italy; 7 Policlinico S. Orsola-Malpighi, SSD Oncologia Medica Addarii, Bologna, Italy; 8 AO Ordine Mauriziano, SCDU Ginecologia ed Ostetricia, Torino, Italy; 9 ASST Fatebenefratelli Sacco, UOC di Oncologia, Milano, Italy; 10 Istituto di Ricerche Farmacologiche Mario Negri IRCCS, Milano, Italy

**Keywords:** Gynaecological cancer, Ovarian cancer

## Abstract

**Background:**

Trabectedin, in addition to its antiproliferative effect, can modify the tumour microenvironment and this could be synergistic with bevacizumab. The efficacy and safety of trabectedin and bevacizumab ± carboplatin have never been investigated.

**Methods:**

In this phase 2 study, women progressing between 6 and 12 months since their last platinum-based therapy were randomised to Arm BT: bevacizumab, trabectedin every 21 days, or Arm BT+C: bevacizumab, trabectedin and carboplatin every 28 days, from cycles 1 to 6, then trabectedin and bevacizumab as in Arm BT. Primary endpoints were progression-free survival rate (PFS-6) and severe toxicity rate (ST-6) at 6 months, assuming a PFS-6 ≤35% for BT and ≤40% for BT+C as not of therapeutic interest and, for both arms, a ST-6  ≥ 30% as unacceptable.

**Results:**

BT+C (21 patients) did not meet the safety criteria for the second stage (ST-6 45%; 95%CI: 23%–69%) but PFS-6 was 85% (95%CI: 62%–97%). BT (50 patients) had 75% PFS-6 (95%CI: 60%–87%) and 16% ST-6 (95%CI 7%–30%).

**Conclusions:**

BT compared favourably with other platinum- and non-platinum-based regimens. The combination with carboplatin needs to be assessed further in a re-modulated safer schedule to confirm its apparent strong activity.

**Clinical Trial Registration:**

NCT01735071 (Clinicaltrials.gov).

## Background

The burden of deaths from ovarian carcinoma (5% of all cancer deaths) is still well ahead of other cancer sites such as leukaemia, non-Hodgkin lymphoma, uterine corpus, liver and brain^1^. Despite the initial good response to complete surgical cytoreduction followed by paclitaxel and platinum-based chemotherapy, most patients still face relapse eventually. The time since last platinum chemotherapy gives a continuum of probability of response to further chemotherapy. Disease that relapses between 6 and 12 months after completion of platinum-based chemotherapy is usually deemed “partially platinum-sensitive” and is a field of intense clinical investigation.

Partially platinum-sensitive tumour relapses amounts to about 23% of all relapses.^2^ These patients may respond again to platinum-based therapy, but repeated use can be limited by resistance or intolerance.^3^ Trabectedin is an antineoplastic agent with a unique mechanism of action. It binds covalently to the minor groove of DNA and disrupts transcription regulation, leading to G2-M cell cycle arrest and ultimately apoptosis.^4^ Preclinical studies indicate that trabectedin, besides having a potent antiproliferative effect, modifies the tumour microenvironment by reducing the number and function of macrophages in the tumour stroma (tumour associated macrophages—TAM). Since TAM produce several angiogenic factors, their inhibition by trabectedin results in an anti-angiogenic effect that is demonstrated well in preclinical systems.^5,6^

Bevacizumab is an antibody that binds and inactivates VEGF, the main factor that hypoxic cancer cells produce to stimulate endothelial cell proliferation and angiogenesis. The mechanisms of resistance to bevacizumab are not fully clear yet but it seems likely that the reduced function of VEGF pathways is compensated by overproduction of other angiogenic factors.^3^ The combination of compounds that inhibit several angiogenic pathways could, therefore, be advantageous for a prolonged antiangiogenic effect and the combination of trabectedin with bevacizumab inhibit several of these mechanisms at the same time.

Preclinical studies in ovarian cancer xenografts have shown synergism between platinum complexes and trabectedin.^7^ A phase 1 study in patients with advanced solid tumours showed that the combination is feasible and the recommended dose in carboplatin-pretreated patients is carboplatin AUC 4 and trabectedin 0.8 mg/m^2^ every 4 weeks.^8^

The partially platinum-sensitive population enabled us to explore though not formally comparing the efficacy and safety profile of two new chemotherapic regimens: a platinum-free regimen with trabectedin and bevacizumab [BT arm] and a platinum-based triplet with carboplatin, trabectedin and bevacizumab [BT+C arm].

## Methods

### Study design and patients

This multicentre, randomised, non-comparative phase 2 study aimed at assessing the efficacy and safety of two regimens according to a Bryant and Day two-stage design.^9^ Eligible patients satisfied these inclusion criteria: cytological or histological diagnosis of epithelial ovarian cancer (including cancer of the fallopian tube and primary peritoneal cancer); progression-free interval 6-12 months (from the first day of the last cycle of the most recent platinum-based chemotherapy); one or two previous platinum-based chemotherapy lines; measurable or evaluable disease, as defined by Response Evaluation Criteria in Solid Tumors, version 1.1 (RECIST 1.1); life expectancy >12 weeks; ECOG performance status ≤2; no contraindications to dexamethasone or its equivalent as a premedication for trabectedin. As specified by the International Conference on Harmonisation/Good Clinical Practice (ICH/GCP), patients must give signed written informed consent before randomisation.

The study was conducted in accordance with the principles of the Declaration of Helsinki.

Main exclusion criteria were: prior exposure to trabectedin; prior progression while on therapy containing bevacizumab or other VEGF pathway-target therapy; peripheral neuropathy grade ≥2; history of other malignancies; central nervous system metastases; uncontrolled hypertension or clinically significant cardiovascular disease, HCV positivity; history of bowel obstruction (including sub-occlusive disease, related to the underlying disease and history of abdominal fistula, gastrointestinal perforation or intra-abdominal abscess); inadequate haematological, renal, bone marrow, or liver function.

### Treatment plan

#### Patients entering the BT arm received trabectedin

1.1 mg/m^2^, into a central venous catheter as a 3-h infusion on day 1 of a 21-day treatment cycle. Premedication for trabectedin was 8 mg oral dexamethasone the day before trabectedin plus 12 mg iv 30 min before, plus 4 mg per os 24, 48 and 72 h post-infusion. Patients received bevacizumab 15 mg/kg iv on day 1 every 21 days.

From cycles 1–6, patients in the BT+C arm, received carboplatin AUC 4 on day 1 every 28 days, trabectedin 0.8 mg/m^2^ on day 1 every 28 days, bevacizumab 10 mg/kg on days 1 and 15. From cycle 7 treatment was as for BT arm.

Treatment was continued until progressive disease, major toxicity, patient’s intolerance or unwillingness to continue treatment, or at the physician’s discretion.

### Evaluation of disease

Physical and radiological examinations were repeated before the start of treatment and every 12 weeks from enrolment until disease progression or death. Disease assessments were based on RECIST 1.1 criteria

### Safety

All patients were evaluated clinically and with standard laboratory tests before and at each cycle. All adverse events (AEs), graded according to the National Cancer Institute-Common Terminology Criteria for Adverse Events (NCI CTCAE), version 4.0, were reported until 30 days from the last dose of the study drugs. Serious adverse events (SAE) and other adverse events (AE) believed to be related to study drugs were reported afterwards.

### Statistical analysis

Allocation to treatment arm was done centrally using a computer-generated permutated block randomisation procedure with a 1:1 ratio and stratifying by centre. According to Bryant and Day design, two primary endpoints were defined: the rate of patients alive and progression-free at 6 months from study entry [PFS-6] and the rate of patients with severe toxicity within 6 months from study entry (ST-6). The following three conditions were considered as ST-6 events: (a) absolute neutrophil count (ANC) <0.5 × 10^9^/L lasting >7 days or with fever, (b) platelets <25 × 10^9^/L, (c) any other NCI-CTCAE grade 3–4 non-haematological toxicities that caused permanent interruption of one or more drugs. Primary endpoints were assessed in the per-protocol (PP) population that included all patients with no major violations of eligibility criteria who had received at least 12 weeks of treatment (unless all treatment drugs were interrupted sooner for progressive disease, toxicity or death). Women who had not progressed or died before 6 months and without a disease evaluation between week 22 and the 27 were not classified as progression-free and were not considered evaluable for the primary analysis, unless the absence of disease progression was confirmed in the disease evaluations after week 27. PFS-6 and ST-6 were provided with their 80% and 95% confidence intervals.

For the BT arm we assumed that 35% PFS-6 (p_0_) or less would mean the combination was of no therapeutic interest. For the BT drug combination to deserve further development we set a target PFS-6 of 60% (p_1_).

For the BT+C arm we took PFS-6 40% at p_0_ = and 65% at p_1_ = . As regards the toxicity endpoint, for both the doublet and triplet combinations a ST-6 of 30% or more would be considered unacceptable, while severe toxicity 10% or less would be considered promising. Type I and type II error probabilities were set at 10%, one-sided and 10% (power = 90%).

**Fig. 1:**
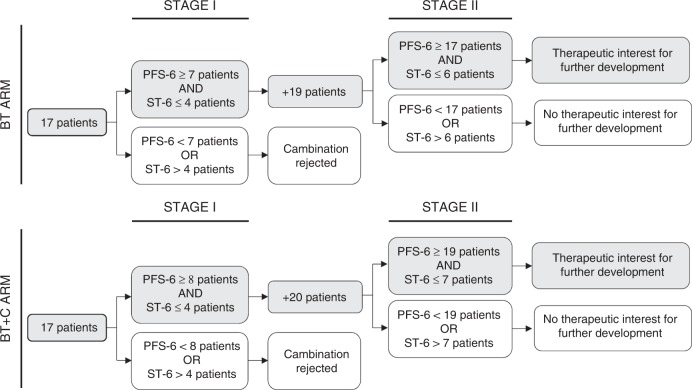
Study design (BT arm: Bevacizumab + Trabectedin. BT+C arm: Bevacizumab + Trabectedin + Carboplatin. PFS-6: progression-free survival at 6 months. ST-6: severe toxicity at 6 months)

The study was divided into two stages, each with pre-set decisional thresholds for efficacy and toxicity (see Fig. 1).

Secondary objectives of the study were progression-free survival (PFS), overall survival (OS), and the toxicity profile. The secondary efficacy endpoints were evaluated in the PP population. PFS was defined as the time between study entry and progression or death for any cause. Subjects with no recurrence or who died while on study were censored at the last disease assessment date. OS was defined as the time between the study entry and death, regardless of the cause of death. Subjects who were not reported as having died at the time of the analysis were censored at the date they were last known to be alive. Survival curves were estimated by the Kaplan–Meier (KM) method.

The toxicity profile was evaluated in the safety population that included all patients without major violations of the eligibility criteria who had received at least one treatment dose. For any single toxicity, the absolute and relative frequencies of events and the highest grade experienced by each subject were provided. Continuous variables were expressed as medians with their interquartile range (IQR). All analyses were done with SAS software, versions 9.4 (SAS Institute).

## Results

In all 71 women with partially platinum-sensitive recurrent ovarian cancer entered this trial from eight Italian centres.

### BT arm

**Fig. 2:**
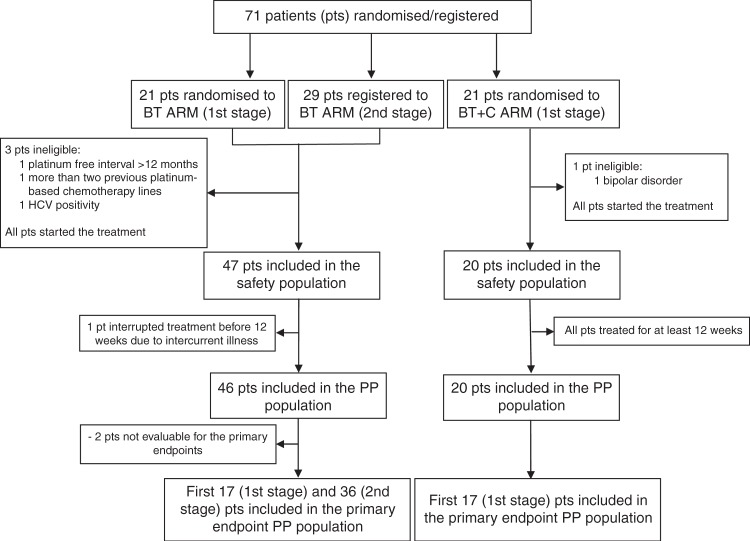
Patients flow-chart (BT arm: Bevacizumab + Trabectedin. BT+C arm: Bevacizumab + Trabectedin + Carboplatin. PP: per-protocol)

From July 2013 through December 2016, 50 patients were randomised/enroled in the BT arm. Figure 2 shows the patients flow-chart and summarises the populations for primary and secondary analyses.

Table 1 shows the clinical characteristics of the 47 patients with no major violations of eligibility criteria. Their median age was 60.8 (IQR: 52.1–69.9) years at study entry; 34 (72%) were facing their first progression of disease, and the others 13 (28%) the second. The median platinum-free interval from the previous platinum-based therapy was 8.9 months (IQR: 7.9–10.0; range 6.0–12.9). At analysis, three patients were still on treatment with BT. Adherence to treatment was satisfactory. Of the 44 patients who had discontinued treatment at the time of analysis, bevacizumab and/or trabectedin had been discontinued for causes independent of disease progression in 17 (39%) and of these, 12(27%) for toxicity or SAE. The median number of BT cycles was 10 (IQR: 6–16; range 2–34); 27/47 (57%) patients received ten or more cycles of BT.

**Table 1:** Tumour characteristics at first diagnosis and prior treatments—patients with no major violations of eligibility criteria

	Bevacizumab + Trabectedin + Carboplatin (20)	Bevacizumab + Trabectedin (47)
*Primary site*		
Ovary	20 (100)	45 (95.7)
Fallopian	0 (0.0)	2 (4.3)
*FIGO stage*		
IIA	1 (5.0)	1 (2.1)
IIB-IIC	0 (0.0)	2 (4.2)
IIIA	0 (0.0)	1 (2.1)
IIIB	0 (0.0)	5 (10.6)
IIIC	16 (80.0)	25 (53.2)
IV	2 (10.0)	11 (23.4)
Unknown	1 (5.0)	2 (4.3)
*Histological grade*		
1–2	0 (0.0)	5 (10.6)
3	20 (100)	38 (80.9)
Unknown	0 (0.0)	4 (8.5)
*Histological type*		
Serous	15 (75.0)	40 (85.1)
Other	4 (20.0)	7 (14.9)
Unknown	1 (5.0)	0 (0.0)
*Number of previous chemotherapy lines*		
One	17 (85.0)	34 (72.3)
Two	3 (15.0)	13 (27.7)
*Last platinum-based chemotherapy type*		
Carboplatin	0 (0.0)	2 (4.3)
Carboplatin + Taxol	18 (90.0)	36 (76.6)
Carboplatin + Gemcitabine	1 (5.0)	2 (4.3)
Carboplatin + PLD	1 (5.0)	5 (10.6)
Other platinum-based chemotherapy	0 (0.0)	2 (4.3)
*Surgery after the last progression*		
No	19 (95.0)	45 (95.7)
Yes	1 (5.0)	2 (4.3)

In the PP population (46 patients), 9 (20%) had a complete (CR) and 24 (52%) a partial response (PR). Eight (17%) patients reached stable disease (SD) and 5 (11%) had progressed by the first disease assessment. Efficacy and the main safety results for first and second stages are shown in Table 2.

**Table 2:** Primary analysis of PFS-6 and ST-6 —per protocol population for primary endpoint

	Bevacizumab + Trabectedin + Carboplatin	Bevacizumab + Trabectedin
STAGE I (17 patients in each arm)		
PFS rate at 6 months (PFS-6)—no. (%)	14 (82.4)	10 (58.8)
80%CI	65% to 93%	41% to 75%
Severe toxicity at 6 months (ST-6)—no. (%)	8 (47.1)	4 (23.5)
80%CI	30% to 65%	11% to 42%
ANC <0.5 × 10/L >7 days and/or fever	4 (50.0)	0 (0.0)
Platelets <25 × 10/L	4 (50.0)	0 (0.0)
Any other grade 3–4 non-haematological toxicities that required permanent interruption of one or both drugs	2 (25.0)	4 (75.0)
STAGE II (36 patients)		
PFS rate at 6 months (PFS-6) –no. (%)	–	25 (69.4)
80%CI		58% to 80%
Severe toxicity at 6 months (ST-6) –no. (%)	–	6 (16.7)
80%CI		9% to 27%
ANC <0.5 × 10/L >7 days and/or fever	–	0 (0.0)
Platelets <25 × 10/L	–	1 (16.7)
Any other grade 3–4 non-haematological toxicities that required permanent interruption of one or both drugs	–	5 (83.3)

*80%CI* 80% confidence interval, *ANC* absolute neutrophil count

Of all the 44 patients evaluable for PFS-6, 11 (25%) progressed and 33 were alive and progression-free 6 months after study entry, so the PFS-6 was 75% (95%CI: 60% to 87%). Seven of the 44 (16%; 95%CI: 7% to 30%) patients experienced ST-6 events.

**Fig. 3:**
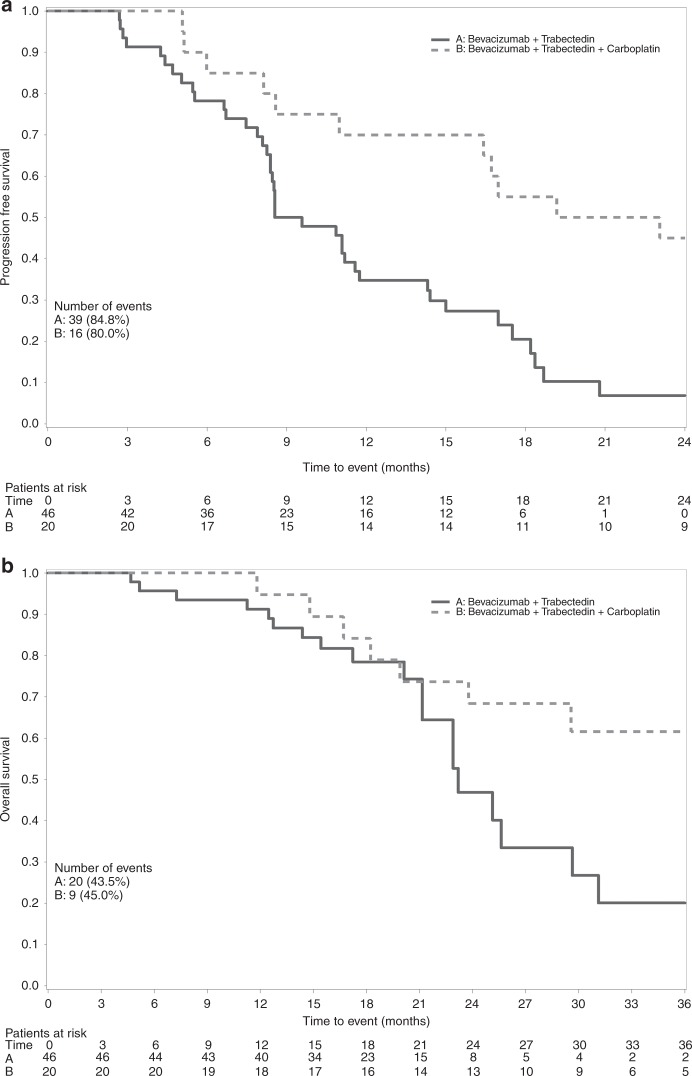
Kaplan–Meier plot of progression-free survival (**a**) and overall survival (**b**) in the per-protocol population

After a median follow-up of 20.7 months, 39 (85%) patients had progressed or died. Median PFS was 9.1 months (IQR: 6.7–17.0). Figure 3a shows the KM curves of PFS. During the study, 20 (44%) patients died. Median OS was 23.2 months (IQR: 20.1–31.1). Figure 3b shows the KM curves for OS.

The BT safety population included 47 patients. There were 1092 AEs, 703 grade 1 (64%), 277 grade 2 (25%), 99 grade 3 (9%), 10 grade 4 (1%) and 1 grade 5 (<1%); 83%of patients had at least one grade ≥3 AE (Table S1). Eleven SAEs occurred in 11 patients (23%). One of these was fatal as the patient died of bowel perforation 30 days after the sixth course of BT. The clinician reported a causal relation with bevacizumab. The patient also had peritoneal carcinosis.

### BT+C arm

From July 2013 through March 2016, 21 patients were randomised to the BT+C arm. Figure 2 shows the flow-chart and the populations for primary and secondary analyses. Table 1 lists the clinical characteristics of the patients with no major violations of eligibility criteria. Median age was 59.4 years (range 54.0–66.1) at study entry; 17 (85%) patients were facing their first progression of disease and the other 3 (15%) the second. The median platinum-free interval from the previous platinum-based therapy was 8.2 months (IQR: 6.6–10.7; range 6.1–12.8).

Adherence to treatment was problematic for 8/20 patients (40%) who stopped carboplatin before six cycles while continuing with BT. Five of these eight had an allergic reaction to carboplatin and three stopped carboplatin because of haematologic toxicity. The median number of carboplatin cycles was 6 (IQR: 2–6; range 1–6). A median of 19 cycles of BT was administered (IQR: 10.5–26.5; range 6–43);16 patients (80%) received ten or more BT cycles. At the time of analysis, one patient was still on treatment with BT.

In the PP population, CR was observed in 9 (45%) and PR in 8 (40%) patients. Three patients (15%) reached SD and none progressed at the first disease assessment.

Efficacy and safety for the first stage are illustrated in Table 2. The BT+C arm was not expanded to second stage on account of excessive toxicity.

None of the 20 evaluable patients in the BT+C arm died; three (15%) progressed before 6 months from randomisation, while 17 were alive and progression-free 6 months from study entry; therefore the PFS-6 was 85% (95%CI: 62%–97%). Nine out of 20 (45%; 95%CI: 23%–69%) experienced ST-6 events.

After a median follow-up of 32.5 months, 14 patients (70%) progressed, 9 (45%) died and 16 (80%) progressed or died. Median PFS and OS were respectively 21.1 months (IQR: 9.8–29.6) and 42.6 months (first quartile 19.9, third quartile not reached), respectively. Figure 3 shows the KM curves for PFS (panel a) and OS (panel b).

The BT+C safety analysis population comprises 20 patients. There were 783 AEs, 479 grade 1 (61%), 211 grade 2 (27%), 70 grade 3 (9%), 20 grade 4 (3%), and 3 grade unknown. The incidence of patients with at least one grade 3 or 4 AE was 90%. Table S2 lists the numbers of patients with at least one grade 3 or 4 specific AEs. Seven SAEs occurred in 7 patients (35%). One of these was fatal as the patient died of acute leukaemia. In this patient carboplatin AUC 4 had to be reduced to AUC 3 and trabectedin from 0.8 to 0.6 mg/m^2^ because of bone marrow toxicity during the first six cycles but then the bone marrow recovered, and the acute leukaemic blast crisis occurred after a further 28 BT cycles.

## Discussion

The partially platinum-sensitive ovarian cancer patients in the present investigation, are part of a unique population for whom either platinum-based or non-platinum-based treatments may be indicated. Testing non-platinum-based new regimens is a clinical need for patients who cannot receive further platinum after front-line chemotherapy because of toxicity or intolerance.

The artificial prolonging of the platinum-free interval with a non-platinum-based chemotherapy that aims at boosting the efficacy of later platinum rescue is another intriguing rationale that has been crossing clinical oncology for some time.^10^ The recently published MITO 8 randomised clinical trial failed to support this mechanism of action: the sequence of non-platinum followed by platinum did not show any PFS and OS benefit over platinum first, in the partially platinum-sensitive patients.^11^ However, the non-platinum-based regimen in the MITO 8 trial consisted of single-agent pegylated liposomal doxorubicin (PLD), which was probably underperforming. Thus, the hypothesis will be definitively confirmed or refuted by the INOVATYON trial which randomised patients to a non-platinum-based doublet (trabectedin/PLD, followed by platinum at progression) or to a platinum-based doublet (carboplatin/PLD) (ClinicalTrials.gov identifier (NCT number): NCT01379989). The use of trabectedin in the non-platinum regimen is interesting because of the strong preclinical evidence that this drug may enhance the efficacy of subsequent treatments with platinum compounds.^10,12^

The specific design of the present phase 2 study enabled us to evaluate two treatment regimens in terms of both efficacy (PFS-6) and safety (ST-6) at the same time. Furthermore, randomisation created two similar patient populations on which to assess the two treatment regimens with a single study protocol and avoiding any selection bias.

The non-platinum-based doublet of trabectedin and bevacizumab met the protocol criteria for efficacy and safety at 6 months with PFS-6 over 35% and severe toxicity below 30%. The median PFS of BT was 9.1 months which compares favourably with the non-platinum-based regimens (trabectedin + PLD: 7.4 months,^13^ PLD: 5.5 months^13^ and 5.0 months^11^) and even with platinum-based ones (carboplatin + PLD: 9.4 months,^14^ carboplatin + taxol: 8.8 months,^14^ platinum-based: 9.0 months^11^) that have been tested in this restricted partially-platinum-sensitive population. The safety of BT was satisfactory and manageable and there was no evidence of synergistic toxicity between these two drugs.

Testing new platinum-based regimens in the partially platinum-sensitive ovarian cancer population merits priority, considering the dismal prognosis of these patients whose survival has remained around 20 months since the introduction of platinum in the 70’s. Our platinum-based triplet of carboplatin, trabectedin and bevacizumab was tested only in the first stage of this trial because its toxicity exceeded the pre-set threshold of 30% and we could not expand the cohort to the second stage. Grade 3–4 haematologic toxicity and grade 3–4 non haematological toxicities, which required permanent interruption of the drugs, hampered the continuation of the trial. The high frequency of hypersensitivity reactions to carboplatin (5 patients) was also unexpected: it might be related to the large number of prior carboplatin courses or, alternatively, to some interaction with the trabectedin. The latter hypothesis is unlikely, particularly considering the patients” pre-treatment with corticosteroids. However, 12 out of 20 patients (60%) were still able to receive the planned six cycles of carboplatin and 16 managed ten or more cycles of BT. The BT+C regimen appeared very active considering that all patients enjoyed some clinical benefit and the median PFS and OS were 21.1 and 42.6 months. Although the BT+C sample was small, so no firm conclusions can be drawn, we think that this activity signal should be picked up while developing a more manageable schedule. The possible synergy between trabectedin, bevacizumab and carboplatin could translate into clinical reality wherever other platinum combinations fail to improve on the efficacy of the standard carboplatin+paclitaxel regimen, even in the front-line setting.^15^ Finally, our trial was run in a bevacizumab-naive population but the recently presented MITO16B-MaNGO-OV2 randomised trial showed that re-challenge with bevacizumab in ovarian cancer relapse after front-line chemotherapy plus bevacizumab confers a significant PFS benefit in comparison with the same treatment without bevacizumab.^16^ This can extend treatment possibilities for the BT regimen.

Limitations of our study include those inherent to phase 2, such as a small sample size, early primary endpoints (PFS-6 and ST-6) and, in our case, the lack of a parallel comparison or calibration group. Nonetheless, our indirect comparisons for the BT arm seem robust enough as the patient populations compared seem homogenous in terms of principal confounding variables, such as platinum-free interval, histologic type and number of previous chemotherapy lines.^11,13,14^ Another potential study limitation relates to the fact that we did not collect the information about germinal or somatic BRCA status. Previous indirect observations and a recent sub-group analysis of the OVC-3006 randomised trial support a possible synergistic effect of BRCA mutation with trabectedin.^17,18^

## Conclusions

The BT regimen showed worth-while clinical activity and limited toxicity that can position it as a therapeutic opportunity for women with partially platinum-sensitive disease. The apparent good activity of the BT+C regimen warrants further studies with a re-modulated schedule to limit its toxicity.

## Supplementary information

Table S1 and table S2

## Data Availability

Data supporting the results reported can be found at Mario Negri Institute IRCCS for Pharmacologic Research, Milan. Data sharing is encouraged, and data are available on request to the corresponding author.
