# Supplementary material for: Multicenter, randomised, open-label, non-comparative phase 2 trial on the efficacy and safety of the combination of bevacizumab and trabectedin with or without carboplatin in women with partially platinum-sensitive recurrent ovarian cancer
Source: Br J Cancer. 2019 Sep 20;121(9):744–50. doi: 10.1038/s41416-019-0584-5 (PMC6888836; doi:10.1038/s41416-019-0584-5)
Supplement: Supplementary file 1 — Table S1 and table S2 [file 41416_2019_584_MOESM1_ESM.docx]

**Table S1.** Adverse events with at least one case of grade ≥ 3 - Safety population enrolled in the BT arm (47 patients)

|  | **G1+G2 no. (%)** | **G3 no. (%)** | **G4 no. (%)** | **G3+G4+G5 no. (%)** |
| --- | --- | --- | --- | --- |
| **Anemia** | 20 (42.5) | 2 (4.3) | 0 | 2 (4.3) |
| **Lymphocyte count decreased** | 7 (14.9) | 6 (12.8) | 0 | 6 (12.8) |
| **Neutrophil count decreased** | 8 (17.1) | 14 (29.8) | 4 (8.5) | 18 (38.3) |
| **Platelet count decreased** | 4 (8.6) | 1 (2.1) | 1 (2.1) | 2 (4.3) |
| **White blood cell decreased** | 22 (46.8) | 3 (6.4) | 0 | 3 (6.4) |
| **Bowel obstruction** | 0 | 0 | 1 (2.1) | 1 (2.1) |
| **Bowel perforation** | 0 | 0 | 0 | 1 (2.1)* |
| **Hemorrhoidal hemorrhage** | 0 | 1 (2.1) | 0 | 1 (2.1) |
| **Hemorrhoids** | 1 (2.1) | 1 (2.1) | 0 | 1 (2.1) |
| **Mucositis** | 3 (6.4) | 2 (4.3) | 0 | 2 (4.3) |
| **Fatigue** | 27 (57.4) | 5 (10.6) | 0 | 5 (10.6) |
| **Hepatotoxicity (not specified)** | 5 (10.7) | 2 (4.3) | 0 | 2 (4.3) |
| **Alanine aminotransferase increased** | 15 (31.9) | 4 (8.5) | 0 | 4 (8.5) |
| **Aspartate aminotransferase increased** | 14 (29.8) | 1 (2.1) | 0 | 1 (2.1) |
| **Blood bilirubin increased** | 4 (8.5) | 1 (2.1) | 0 | 1 (2.1) |
| **GGT increased** | 7 (14.9) | 4 (8.5) | 0 | 4 (8.5) |
| **Urinary tract infection** | 1 (2.1) | 1 (2.1) | 0 | 1 (2.1) |
| **Vascular access complication** | 0 | 1 (2.1) | 0 | 1 (2.1) |
| **Weight gain** | 0 | 1 (2.1) | 0 | 1 (2.1) |
| **CPK increased** | 0 | 0 | 2 (4.3) | 2 (4.3) |
| **Dysarthria** | 0 | 1 (2.1) | 0 | 1 (2.1) |
| **Neurotoxicity** | 1 (2.1) | 1 (2.1) | 0 | 1 (2.1) |
| **Seizure** | 0 | 1 (2.1) | 0 | 1 (2.1) |
| **Syncope** | 1 (2.1) | 1 (2.1) | 0 | 1 (2.1) |
| **Proteinuria** | 4 (8.5) | 3 (6.4) | 0 | 3 (6.4) |
| **Dyspnea** | 2 (4.3) | 1 (2.1) | 0 | 1 (2.1) |
| **Obstructive pulmonary disease** | 0 | 1 (2.1) | 0 | 1 (2.1) |
| **Hypertension** | 19 (40.4) | 5 (10.6) | 1 (2.1) | 6 (12.8) |
| **Thromboembolic event** | 2 (4.3) | 1 (2.1) | 0 | 1 (2.1) |
| * One G5 adverse event occurred. | | | | |

**Table S2.** Adverse events with at least one case of grade ≥ 3 - Safety population enrolled in the BT+C arm (20 patients)

|  | **G1+G2 no. (%)** | **G3 no. (%)** | **G4 no. (%)** | **G3+G4**  **no. (%)** |
| --- | --- | --- | --- | --- |
| **Anemia** | 13 (65.0) | 1 (5.0) | 0 | 1 (5.0) |
| **Febrile neutropenia** | 0 | 0 | 1 (5.0) | 1 (5.0) |
| **Lymphocyte count decreased** | 2 (10.0) | 3 (15.0) | 0 | 3 (15.0) |
| **Neutrophil count decreased** | 5 (25.0) | 2 (10.0) | 7 (35.0) | 9 (45.0) |
| **Platelet count decreased** | 7 (35.0) | 3 (15.0) | 5 (25.0) | 8 (40.0) |
| **White blood cell decreased** | 6 (30.0) | 5 (25.0) | 1 (5.0) | 6 (30.0) |
| **Heart failure** | 0 | 0 | 1 (5.0) | 1 (5.0) |
| **Left ventricular systolic dysfunction** | 0 | 1 (5.0) | 0 | 1 (5.0) |
| **Mucositis oral** | 7 (35.0) | 1 (5.0) | 0 | 1 (5.0) |
| **Alanine aminotransferase increased** | 7 (35.0) | 1 (5.0) | 0 | 1 (5.0) |
| **GGT increased** | 4 (20.0) | 2 (10.0) | 0 | 2 (10.0) |
| **Allergic reaction** | 3 (15.0) | 2 (10.0) | 0 | 2 (10.0) |
| **Hypocalcemia** | 0 | 1 (5.0) | 0 | 1 (5.0) |
| **Hypokalemia** | 0 | 1 (5.0) | 0 | 1 (5.0) |
| **Hypomagnesemia** | 0 | 1 (5.0) | 0 | 1 (5.0) |
| **Hyponatremia** | 0 | 1 (5.0) | 0 | 1 (5.0) |
| **CPK increased** | 2 (10.0) | 0 | 1 (5.0) | 1 (5.0) |
| **Hypertension** | 6 (30.0) | 7 (35.0) | 0 | 7 (35.0) |
